# SARS-CoV-2 Infection and Antiviral Strategies: Advances and Limitations

**DOI:** 10.3390/v17081064

**Published:** 2025-07-30

**Authors:** Vinicius Cardoso Soares, Isabela Batista Gonçalves Moreira, Suelen Silva Gomes Dias

**Affiliations:** 1 Laboratory of Immunopharmacology, Oswaldo Cruz Institute (IOC), Oswaldo Cruz Foundation (Fiocruz), Rio de Janeiro 21040-361, Brazil; isabelabgm.ufrj@gmail.com; 2 Center for Research, Innovation and Surveillance in COVID-19 and Health Emergencies, Oswaldo Cruz Foundation (Fiocruz), Rio de Janeiro 21040-361, Brazil; 3 Program of Immunology and Inflammation, Federal University of Rio de Janeiro UFRJ, Rio de Janeiro 21941-902, Brazil

**Keywords:** SARS-CoV-2, antiviral, COVID-19, variants, repurposing drugs

## Abstract

Since the onset of the COVID-19 pandemic, remarkable progress has been made in the development of antiviral therapies for SARS-CoV-2. Several direct-acting antivirals, such as remdesivir, molnupiravir, and nirmatrelvir/ritonavir, offer clinical benefits. These agents have significantly contributed to reducing the viral loads and duration of the illness, as well as the disease’s severity and mortality. However, despite these advances, important limitations remain. The continued emergence of resistant SARS-CoV-2 variants highlights the urgent need for adaptable and durable therapeutic strategies. Therefore, this review aims to provide an updated overview of the main antiviral strategies that are used and the discovery of new drugs against SARS-CoV-2, as well as the therapeutic limitations that have shaped clinical management in recent years. The major challenges include resistance associated with viral mutations, limited treatment windows, and unequal access to treatment. Moreover, there is an ongoing need to identify novel compounds with broad-spectrum activity, improved pharmacokinetics, and suitable safety profiles. Combination treatment regimens represent a promising strategy to increase the efficacy of treating COVID-19 while minimizing the potential for resistance. Ideally, these interventions should be safe, affordable, and easy to administer, which would ensure broad global access and equitable treatment and enable control of COVID-19 cases and preparedness for future threats.

## 1. Introduction

The emergence of severe acute respiratory syndrome coronavirus 2 (SARS-CoV-2), a novel member of the *Coronaviridae* family, triggered the global outbreak of coronavirus disease 2019 (COVID-19). Its spread was extraordinarily rapid, resulting in a pandemic that imposed unprecedented problems on public health systems, global economies, and social infrastructures [1,2]. Since its initial identification in December 2019, SARS-CoV-2 has been associated with high morbidity and mortality, spreading across continents and overwhelming healthcare systems worldwide.

The continuous emergence of novel viral variants has further complicated containment efforts, caused sustained high transmission rates, and impacted established mitigation strategies. Among these, the Omicron variant has become the predominant circulating strain globally, being distinguished by significantly enhanced transmissibility as well as pronounced immune evasion capabilities [3]. In response to this epidemiological scenario, accelerated efforts have focused on the development and deployment of antiviral therapies and vaccine platforms, which has been supported by extensive experimental and clinical research programs.

Therefore, therapeutic agents have assumed an increasingly crucial role in managing confirmed cases and in reducing the risk of clinical deterioration. Therapeutic strategies that use antivirals are associated with direct action that aims to interfere with different stages of the viral replication cycle. The clinical efficacy of these agents has been demonstrated in numerous trials that were conducted prior to the dominance of the Omicron variant and further evaluated against more recent variants; several antivirals, such as Paxlovid, Veklury, and Lagevrio have received regulatory approval from agencies such as the U.S. Food and Drug Administration (FDA) and the European Medicines Agency (EMA) [4,5,6].

Like other RNA viruses, SARS-CoV-2 undergoes frequent genetic mutations during transmission, which has led to a rapidly evolving profile of circulating variants. These changes not only increase the virus’s adaptability to different hosts and cellular environments but may also contribute to pathogenicity and transmission rates [7,8]. In this context, despite some therapeutic options and the efficacy of current vaccines, the possibility of the emergence and spread of the new viral variants that are resistant to available antivirals or capable of evading the immune response stimulated by vaccines reinforces the need for constant evaluations of the efficacy of the approved antivirals against new variants of SARS-CoV-2, as well as the development of new potential antiviral drugs.

This review aims to present an updated synthesis of the main therapeutic strategies employed against SARS-CoV-2, with a particular focus on the antiviral agents that have shaped clinical management in recent years, their limitations, and the ongoing advances in the development of a range of promising antiviral compounds that act at different stages of the SARS-CoV-2 replication cycle.

## 2. SARS-CoV-2: Structure and Replication

SARS-CoV-2 is a positive-sense single-stranded RNA virus with a genome of approximately 29,900 nucleotides, encoding 29 genes. It encodes 16 nonstructural proteins (NSPs) and 4 structural proteins: spike (S), envelope (E), membrane (M), and nucleocapsid (N) proteins. The viral S glycoprotein binds to the angiotensin-converting enzyme 2 (ACE2) receptor, which is the same receptor that SARS-CoV uses to enter human cells [9,10]. The S protein is composed of two subunits: the S1 subunit, which contains the receptor-binding domain (RBD), and the S2 subunit, which facilitates the fusion of the virus with the host-cell membrane following its primary cleavage by transmembrane serine protease 2 (TMPRSS2). After the proteolytic activation of TMPRSS2, the fusion peptide is exposed, which allows viral entry. Although other sections of the S protein also trigger neutralizing activity, the RBD region is the principal target of neutralizing antibodies and cytotoxic lymphocytes [10,11,12].

Once the genomic RNA (gRNA) is released into the host-cell cytoplasm, replication begins. Ribosomes are rapidly recruited to initiate the translation of the open reading frames (ORFs). These ORFs encode the non-structural polyproteins pp1a and pp1ab, which are cleaved by the viral main protease (Mpro) and the papain-like protease (PLpro) into 16 mature NSPs [10,13]. During this stage, NSP1 plays a key role in suppressing host-mRNA translation, while other NSPs—including NSP12, which contains the RNA-dependent RNA polymerase (RdRp) domain—assemble into replication-transcription complexes (RTCs) within double-membrane vesicles derived from the endoplasmic reticulum (ER) [14]. These RTCs are responsible for the synthesis of full-length strands of complementary negative-sense gRNA, along with a set of negative-sense subgenomic RNAs (sgRNAs) derived from the 3′-proximal region of the viral genome, which serve as templates to produce structural proteins [15]. The complementary negative-sense gRNA is subsequently transcribed back into positive-sense genomic RNA, which undergoes capping mediated by the methyltransferase activity of NSP14 and thus enables the next stages of virion assembly [14].

Simultaneously, negative-sense sgRNAs serve as templates for the synthesis of a set of positive-sense subgenomic messenger RNAs (sg-mRNAs), each of which encodes structural and accessory proteins (ORF2–9b) [16]. The resulting structural proteins, including S, M, and E proteins, are integrated into the lipid bilayer of the ER. These components are then trafficked to the ER–Golgi intermediate compartment (ERGIC), where viral assembly occurs. During this process, N proteins associate with the membrane-inserted structural proteins, promoting the budding of mature virions. These newly formed viral particles acquire a lipid envelope enriched with the S protein, which enables their release from the host cell via exocytosis [10,14].

## 3. Main Targets of Antiviral Drugs Against SARS-CoV-2

**Figure 1:**
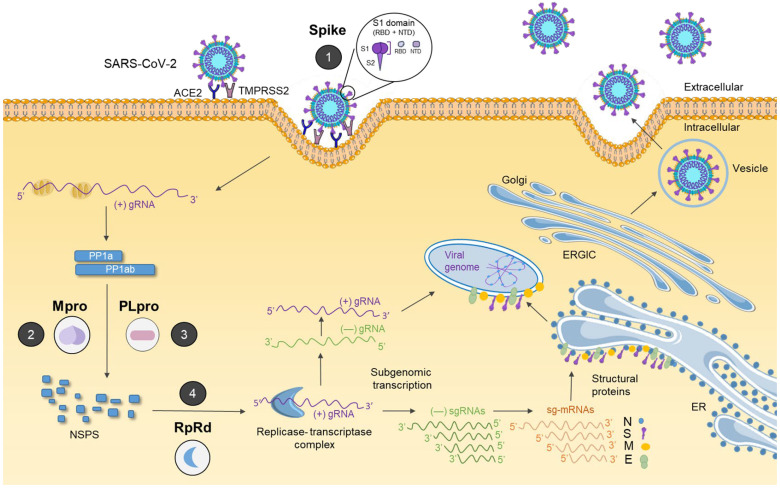
Participation of major antiviral targets throughout the SARS-CoV-2 life cycle. (1) S protein mediates viral attachment to the host ACE2 receptor and is primed by TMPRSS2, promoting the viral entry process. It contains the receptor-binding domain (RBD) and the N-terminal domain (NTD), both located in the S1 subunit, which are primary targets of neutralizing antibodies and antiviral agents aimed at blocking viral entry. (2) Upon entry, viral RNA is released and translated into polyproteins pp1a and pp1ab, which are cleaved by Mpro, which generates mature nonstructural proteins (NSPs). Mpro is a central target of replication-inhibiting antivirals. (3) PLpro, another viral protease, also processes pp1a/pp1ab and plays a dual role by suppressing the host immune response through the removal of ubiquitin and ISG15 from host proteins. Its immunomodulatory function makes PLpro an attractive therapeutic target. (4) RdRp, a central component of the replicase-transcriptase complex, is responsible for synthesizing both subgenomic and genomic RNAs required to produce structural proteins and the assembly of new virions. RdRp is a key enzymatic target for antiviral drugs that inhibit viral RNA synthesis [17,18,19,20].

To better understand the mechanism of action of drugs against SARS-CoV-2, it is essential to understand the replication cycle of the virus, particularly its entry/invasion, replication, genome assembly, and release. These steps represent critical targets for therapeutic interventions and serve as a foundation for the development of potential antiviral drugs for the treatment of COVID-19. In this context, four main viral targets have been identified for the development of antiviral drugs (Figure 1).

The dependence of SARS-CoV-2 on the S protein to obtain entry into the host cells makes it a compelling therapeutic target. The S protein, cleaved by a furin-like protease, consists of two subunits, S1, which binds to host cell receptors, and S2, which mediates fusion between the viral and host membranes, an essential step for infection. The RBD, located in the S1 subunit, facilitates the interaction between the S protein and the ACE2 receptor, allowing the virus to enter host cells [17]. Consequently, inhibition of the RBD–ACE2 interaction has become a key strategy for therapeutic intervention. However, the high mutation rate observed in emerging variants has increasingly limited the clinical efficacy of such approaches, requiring the continuous development of new monoclonal antibodies (mAbs) that target the RBD of the S protein (Figure 1).

The SARS-CoV-2 S protein is highly susceptible to mutations, with notable increases having been observed in current SARS-CoV-2 variants, potentially compromising vaccine efficacy [21]. As a result, researchers are exploring alternative antiviral targets, with enzymes emerging as promising candidates due to the rarity of mutations that affect their active sites. In this context, Mpro has emerged as an interesting target for antiviral drugs, as it is capable of cleaving large polyproteins into functional viral proteins, which are required for viral replication. Inhibiting Mpro activity can disrupt viral replication [18] and represents an effective strategy to combat SARS-CoV-2 (Figure 1).

Another key enzyme in the SARS-CoV-2 replication machinery is PLpro, which, along with Mpro, is capable of cleaving viral polyproteins and host-cell proteins, which makes it a possible antiviral target (Figure 1). Inhibiting PLpro activity may prevent viral replication and reduce viral dissemination within the host. Targeting peripheral sites of PLpro may be a promising antiviral strategy [19].

Finally, RdRp (also known as NSP12), represents one of the primary targets for antiviral drugs against SARS-CoV-2 (Figure 1) [20]. The inhibition of RdRp has been effective in treating several viral infections, including dengue virus, hepatitis C virus, zika virus, chikungunya virus, and influenza. Although RdRp shares structural elements, such as motifs and domains, with DNA and RNA polymerases, each RdRp inhibitor may interact differently with its binding site [22]. These interactions often involve shared structural features or binding conformations, which contribute to the broad-spectrum antiviral activity observed across all RNA virus families.

## 4. Antiviral Therapy Approved for COVID-19

RNA viruses inherently exhibit higher mutation rates than DNA viruses [23,24], and SARS-CoV-2 is no exception. Amino acid substitutions, particularly in surface glycoproteins, can significantly influence the pathogenicity, immune evasion, and vaccine efficacy of viruses, as previously observed in viruses such as chikungunya, ebola, and influenza [25,26,27]. The SARS-CoV-2 genome continues to undergo genetic variation, with frequent mutations having been reported in the S protein [28,29]. Emerging SARS-CoV-2 variants have shown reduced sensitivity to convalescent sera and mAbs [30], which raises serious concerns regarding the effectiveness of current prevention strategies [31]. The receptor-binding motif (RBM) within the RBD of the spike protein is both a key target for neutralizing antibodies and one of the most variable regions of the virus [32]. This high evolutionary capacity of the S protein poses challenges for immunity and vaccine efficacy, prompting the continuous refinement of vaccines and therapeutics to address mutations that affect transmissibility, infectivity, pathogenicity, immune escape, and antigenicity [33]. Neutralizing mAbs that specifically target the RBM of the S protein are considered promising antiviral agents capable of preventing viral attachment [34].

Despite the broad availability of prophylactic vaccines against SARS-CoV-2, vaccination alone does not fully prevent infection, especially given the continuous emergence of viral mutations [35,36]. This reality underscores the crucial role of complementary therapeutic strategies, particularly antiviral drugs, in the ongoing management of COVID-19. Accordingly, the development of new antivirals, along with the repositioning of existing ones, remains a critical priority in controlling the disease’s progression, minimizing severe outcomes, and addressing the limitations associated with the viral evolution and immune evasion mechanisms of the virus.

Antivirals can be grouped based on their molecular targets within the viral replication cycle. In the case of coronaviruses, the antivirals available for clinical therapies can be divided into two groups: antivirals against the viral proteases, whose targets are Mpro and PLpro, and antivirals against genome replication, whose target is RdRp. Although several of these antivirals, such as Paxlovid, Veklury, and Lagevrio, already have proven clinical efficacy [4,5,6], the discovery or repositioning of new antivirals is necessary due to the high mutation rate of SARS-CoV-2. Despite this division, it is important to emphasize that these molecules have a therapeutic effect even in vaccinated people, and that their use does not substitute for SARS-CoV-2 vaccines.

### 4.1. Targeting Viral Proteases

The Mpro or 3CLpro is an essential SARS-CoV-2 main protease that plays an important role in viral replication, cleaving polypeptides and generating NSPs that enable the synthesis of sgRNAs and the suppression of the host immune response. Mpro is a three-domain cysteine protease with a catalytic dyad (Cys145-His41). Once the polypeptide binds to the catalytic site, His41 primes Cys145 for nucleophilic cleavage of the polypeptide bond, splitting into 11 specific sites and generating the NSPs [37]. Mpro is a highly conserved viral protein across the *Coronaviridae* family and exhibits structural similarities with other pathogenic viruses, including hepatitis A virus, dengue virus, enteroviruses, and zika virus [38]. Due to its high degree of conservation and functional importance, Mpro is one of the most promising therapeutic targets for COVID-19.

In this context, Pfizer developed an antiviral therapy that combines two existing antiviral inhibitors: nirmatrelvir and ritonavir. Nirmatrelvir is a polypeptide mimetic that binds to the catalytic site of Mpro. The nitrile group of the drug forms a reversible covalent bond with the Cys145 of Mpro, inhibiting proteolytic activity and blocking viral replication [39]. Ritonavir, on the other hand, is a drug that is mainly used in acquired immune deficiency syndrome (AIDS) treatment, as it acts as an inhibitor of the type 1 protease of human immunodeficiency virus (HIV) by binding to the functional site of the enzyme. Although its primary use is in AIDS treatment, ritonavir is also a strong inhibitor of cytochrome P450 isoenzyme A (CYP450–3A4), which blocks the degradation of nirmatrelvir, and thereby enhances its bioavailability and therapeutic efficacy [40]. Studies on the single oral administration of nirmatrelvir demonstrated that the average time of maximum plasma concentration of the drug (Tmax) without ritonavir was from 0.63 h to 1 h. However, when co-administered with ritonavir, the Tmax was 2.75 h, which is nearly a three-fold increase [41]. Based on these findings, the combination therapy, marketed as Paxlovid, received an emergency use authorization in December 2021 and was officially approved by the FDA in May 2023. It is the first-line oral antiviral treatment for mild-to-moderate cases of COVID-19 in adults and pediatric patients of COVID-19 older than 12 years old, and is highly efficient in preventing the progression of the disease in patients who are at high risk of poor prognosis [42].

The first clinical trial about Paxlovid (NCT04960202) has shown a reduction in clinical complications, such as hospitalization and death risk, by about 89% among patients at high risk of progression to severe COVID-19 when it was initially administered within three days of symptom onset [4,42]. Paxlovid was also effective in containing viral replication during the Omicron wave, reducing clinical complications by 44% compared to nontreated individuals who were vaccinated for COVID-19 [4,43]. Although the treatment remains effective in reducing viral replication, even against the Omicron variant, some authors emphasize the importance of the genomic surveillance of Mpro due to the potential development of nirmatrelvir resistance over time [44]. Despite no major safety concerns having been reported, dysgeusia and diarrhea are frequent adverse events, and the treatment is contraindicated in patients with severe renal and hepatic impairment [41,45]. In addition, Paxlovid can cause drug–drug interactions (DDIs) due to the pharmacokinetic enhancer function of ritonavir that can boost not only nirmatrelvir but also other concomitant medications. Patients with cancer, for example, need to receive a reduction in their dose that is between 10% and 75% of the original dose of the small-molecule kinase inhibitors (KIs) treatment to prevent potential life-threatening interactions [46]. Therefore, Paxlovid is a promising oral antiviral drug that is highly recommended for treating COVID-19 but requires careful monitoring for adverse effects and contraindications.

### 4.2. Targeting Genome Replication

RdRp is a crucial viral enzyme that participates in the replication and transcription of SARS-CoV-2, catalyzing the elongation of the viral RNA genome, and has no homolog in host cells. The incorporation of nucleoside triphosphate (NTP) in the catalytic site triggers the translocation of the RNA and clears the binding site for the next incoming NTP. Several research groups have determined that the cryo-EM RdRp has a common structure and function across several viral species, including influenza, hepatitis C virus, zika virus, and coronaviruses (CoVs), owing to its conserved overall structure as well as its active site [47,48]. Thus, antiviral agents acting on the RdRp of other RNA viruses may block the viral replication of SARS-CoV-2. The structural similarity of RdRp makes drug repurpose an effective strategy to shorten the time of drug development. Over the past decade, several pharmaceutical companies have focused on developing RdRp inhibitors, of which the most studied compounds are nucleotide analogs such as ribavirin, sofosbuvir, daclatasvir, dasabuvir, and remdesivir [49,50,51,52].

Remdesivir (Veklury) was the first antiviral drug that received an emergency use authorization for COVID-19 treatment by the FDA, in May 2020. Initially developed against the ebola virus, remdesivir is an intravenous drug recommended for adults and pediatric patients with COVID-19 who are aged 28 days and older, weigh at least 3 kg, and require hospitalization. During clinical trials, hospitalized patients treated with remdesivir had a clinical improvement with a short recovery time of 10 days, compared with the 15 days for patients who received a placebo [53]. Additionally, remdesivir was able to reduce the mortality rate compared to the placebo group, although its efficacy was not superior to that of Paxlovid. Despite there being no safety concerns against the drug, the liver enzymes alanine aminotransferase (ALT) and aspartate aminotransferase (AST) were increased in patients who received remdesivir, which suggests potential hepatotoxicity as the primary adverse effect of the drug’s administration. The elevation of liver enzymes was associated with mild and reversible prothrombin time prolongation, but no clinical evidence of hepatitis was reported [54]. Therefore, like Paxlovid, remdesivir is contraindicated for patients who have severe hepatic impairments.

Remdesivir is a prodrug that becomes active when converted to its triphosphate form GS-441524 (also known as remdesivir triphosphate form, RTP). This active form is recognized by RdRp as a substrate and is incorporated into the viral RNA product chain as remdesivir monophosphate (RMP). Following the incorporation of RMP, RdRp adds three more nucleotides before it stalls, thereby impairing the viral RNA elongation through a delayed chain-termination mechanism [55]. GS-441524 shows broad-spectrum antiviral activity against various pathogenic RNA viruses, including multiple variants of ebola virus, and human CoVs in both cultured cells and animal models [56,57]. The efficacy of remdesivir on SARS-CoV-2 infection revealed that remdesivir had an excellent inhibitory effect on the replication of SARS-CoV-2 [5], which supports the studies of remdesivir in COVID-19 clinical trials.

In December 2021, the FDA also issued an emergency use authorization for another antiviral drug that targets RdRp. Molnupiravir (Lagevrio) is an orally administered antiviral that was developed by Merck for the treatment of mild-to-moderate COVID-19. In contrast to the other antiviral drugs, molnupiravir is an alternative therapy that is recommended only when FDA-authorized treatment options are unavailable or clinical inappropriate. Furthermore, molnupiravir is not recommended for use during pregnancy, as preclinical studies have indicated that the drug exhibits toxicity to embryonic development [58]. During the pivotal phase III of the MOVe-OUT clinical trial, the risk of hospitalization was lower with molnupiravir than with placebo [6]. Also, this antiviral treatment was able to inhibit viral replication and prevent transmission in murine and ferret models [59,60]. In a phase II clinical trial, molnupiravir demonstrated high antiviral efficacy against SARS-CoV-2 [61], leading to the faster clearance of viral RNA and a higher rate of viral elimination compared to placebo. Safety analyses supported the ongoing clinical development of this drug, indicating that molnupiravir was well tolerated with no significant adverse events, which implies that it has potential as an oral agent to reduce the viral replication and progression of COVID-19 in the early stages of the disease. However, the 2022 PANORAMIC randomized trial with vaccinated patients demonstrated no difference in the risk of hospitalization between the two groups, raising questions about the effectiveness of this antiviral in vaccinated patients [62].

Despite targeting the same viral enzyme, molnupiravir and remdesivir exhibit distinct mechanisms of action. The active form of molnupiravir, β-d-N4-hydroxycytidine (NHC) triphosphate, acts as a substrate for RdRp, replacing the original cytidine triphosphate or uridine triphosphate. However, when the synthesized RNA serves as a template for replication, NHC promotes the incorporation of guanine and adenine triphosphates, which results in the generation of mutated RNA. Consequently, molnupiravir increases the frequency of mutagenesis, inducing lethal mutations into the viral RNA genome, which prevents the formation of infectious particles and the inhibition of viral replication [55].

Paxlovid, Veklury, and Lagevrio, antivirals that are approved for treating COVID-19, have advantages and disadvantages (Table 1). Paxlovid stands out for being orally administered and for significantly reducing the risk of hospitalization and mortality rate, although its drug–drug interactions may restrict its use. Remdesivir is more invasive because it is administered intravenously, and it is associated with shorter recovery times for hospitalized patients, but it is not significantly effective in reducing the mortality rate. Molnupiravir, also orally administered, has a lower effectiveness than Paxlovid and there are safety concerns regarding its use during pregnancy. Therefore, it is crucial to consider the patient’s individual profile and clinical status when selecting the most appropriate therapy. Nonetheless, the emergence of specific viral mutations has led to reports of resistance against the three main approved antivirals. The prevention of the emergence and dissemination of antiviral resistance necessitates appropriate clinical management and therapeutic administration supported by timely laboratory testing and effective surveillance strategies.

## 5. The Challenges and Limitations of Approved Antivirals for COVID-19

The ongoing need to develop new antiviral therapies for COVID-19 is justified by several biological and clinical challenges that have become increasingly evident throughout the pandemic. One of the most significant concerns is the continued emergence of SARS-CoV-2 variants. As the virus evolves, it accumulates mutations, particularly in the S protein and nonstructural proteins involved in viral replication, that cause differences in its transmissibility and immunity evasion and that can impact the efficacy of existing antiviral agents [63]. Direct-acting antivirals, such as remdesivir, molnupiravir, and nirmatrelvir/ritonavir, generally maintain their activity by targeting conserved viral elements; however, specific mutations have been reported that could affect the transmissibility and illness severity of the virus and potentially reduce its drug susceptibility. 

In this context, multiple studies have identified mutations associated with resistance to nirmatrelvir/ritonavir through in vitro experimentation. The identification and analysis of the development of antiviral resistance in vitro may serve as a predictor of clinical outcomes. Multiple mutations were found at the S144, M165, E166, H172, and Q192 residues of Mpro, which were found to be more likely to maintain enzymatic activity while also being associated with resistance to nirmatrelvir [64].

Notably, the E166 residue of SARS-CoV-2 Mpro was identified as a critical point for the development of resistance to nirmatrelvir, the key component of Paxlovid. Among the various substitutions observed in vitro, E166V has been shown to confer the highest level of resistance, with approximately a 100-fold reduction in susceptibility to nirmatrelvir. However, this mutation also significantly impairs viral fitness. Interestingly, co-occurring mutations such as L50F or T21I can restore enzymatic activity and compensate for this loss of fitness without reversing the resistance phenotype. Given the structural similarity between valine (E166V) and alanine (E166A), both substitutions exhibit comparable resistance phenotypes, which further emphasizes the importance of the E166 residue in inhibitor binding [65,66,67]. In addition, clinical case reports have previously reported E166V and L50F mutations in immunocompromised patients treated with Paxlovid; however, there was no evidence of viral clearance [68,69].

In addition, mutations such as P132H and K90R, although located outside the active site, have been shown to affect the overall conformation and structural dynamics of Mpro, indirectly compromising the efficacy of nirmatrelvir [67,70]. These findings underscore the potential for antiviral resistance development as well as highlighting the importance of ongoing surveillance and the need for next-generation protease inhibitors.

In contrast, mutations in RdRp (NSP12) are less common, but emerging evidence from both in vitro studies and clinical reports has highlighted the potential for resistance mutations to arise in patients treated with remdesivir [71,72,73]. Different mutations within the RdRp (NSP12) have been associated with reduced susceptibility to the drug, particularly in immunocompromised individuals undergoing prolonged therapy [68].

One such mutation, V166L, was identified in an immunocompromised patient with persistent SARS-CoV-2 infection following remdesivir treatment [68]. In vitro analyses confirmed the emergence of this mutation, which confers a mild reduction in drug susceptibility and may have contributed to a resistance mechanism in this case [74]. Similarly, the E802D mutation in NSP12 has been observed in patients receiving remdesivir, including an elderly immunocompromised individual. Functional studies have demonstrated that E802D and E802A significantly increase the half-maximal inhibitory concentration of remdesivir by six-fold and four-fold, respectively, which suggests that modifications at this position may substantially alter the efficacy of the remdesivir [71,73]. These results indicate that mutations like E802 may reduce remdesivir’s binding affinity by causing minor structural changes in the RdRp active site, potentially altering steric interactions and allowing RNA elongation to continue despite the incorporation of the drug’s active form.

Additionally, the mutation G671S in RdRp (NSP12) has been positively selected and may confer a selective advantage to emerging variants [75]. Furthermore, in a retrospective cohort study, this same mutation was observed after the administration of remdesivir [76]. Other RdRp mutations have also been implicated in resistance. For example, the mutations F480L and V570L interfere with remdesivir’s binding by destabilizing the interface between polymerase domains [77]. Notably, this and other resistance-associated mutations, such as S759A, V792I, and C799F, have been identified following extended in vitro passaging or the analysis of clinical isolates from treated patients, including those infected with Omicron sublineages. These mutations may disrupt the binding of remdesivir and its parent nucleoside GS-441524, compromising their inhibitory activity [78,79].

Despite these findings, the frequency of NSP12 substitutions has been reported to be similar between patients treated with remdesivir and those receiving a placebo [80]. Indeed, resistance to remdesivir appears to require serial passaging under drug pressure, which indicates that such mutations may not readily emerge in typical clinical scenarios [74,78] and suggests that some of these mutations may arise through natural viral evolution rather than drug-driven selection alone.

Unlike remdesivir, which acts through chain termination, molnupiravir exerts its antiviral activity via a mechanism known as lethal mutagenesis. Its active metabolite, NHC, is incorporated into viral RNA in place of cytidine or uridine, which leads to ambiguous base pairing with either guanosine or adenosine. This process introduces random mutations into the viral genome, progressively reducing its integrity and ultimately causing viral extinction due to genomic instability [81]. Because molnupiravir targets the highly conserved RdRp, it has demonstrated broad-spectrum activity and retained antiviral efficacy across a wide range of SARS-CoV-2 variants, including Alpha (B.1.1.7), Beta (B.1.351), Gamma (P.1), Delta (B.1.617.2), Omicron sublineages (BA.1–BA.5, BQ.1.1, XBB.1.5), Lambda (C.37), and Mu (B.1.621). In addition, no phenotypic or genotypic resistance to NHC has been documented, even after 30 serial passages of SARS-CoV-2 in vitro under drug pressure. Viral cultures exposed to NHC showed a random distribution of nucleotide substitutions that was consistent with its mutagenic mechanism, but no emergence of stable resistance mutations was observed [82]. This suggests a low potential for classical resistance development, likely due to the broad, non-specific nature of the drug’s error-inducing activity.

However, some concerns have been raised about the potential for molnupiravir to promote the emergence of transmissible variants, particularly in immunocompromised hosts who may experience prolonged infection and incomplete viral clearance. In this context, the A716V mutation in NSP12 has been detected in some patients receiving molnupiravir. Although this residue lies within the catalytic core of RdRp, no functional evidence has confirmed its role in drug resistance [83]. The impact of such substitutions remains uncertain, but their detection underscores the importance of genomic surveillance during and after treatment, especially in vulnerable populations.

Although concerns have been raised about possible off-target effects or host mutagenicity, current studies support the safety profile of molnupiravir, indicating minimal risk of its incorporation into host DNA and only partial correction of NHC-induced mutations by the viral proofreading exonuclease (ExoN) [81]. Overall, while molnupiravir continues to demonstrate robust antiviral efficacy, particularly against emerging variants, ongoing molecular monitoring is crucial to detect and understand any rare but clinically relevant resistance-associated mutations that may compromise future treatment strategies.

In addition to the potential development of antiviral resistance, the clinical limitations of approved antivirals also highlight the importance of expanding the range of treatments. Paxlovid (nirmatrelvir/ritonavir) is associated with significant drug interactions, which limits its use in patients with complex comorbidities or polypharmacy [46]. Remdesivir requires intravenous administration, which restricts its use, primarily for hospitalized patients, and its use is not recommended in individuals with severe hepatic impairment [53]. Molnupiravir, although available orally, is contraindicated during pregnancy due to potential teratogenicity [58]. Furthermore, these antivirals are most effective when administered early in the course of infection, typically within the first five days of symptom onset, which poses challenges for timely diagnosis and access to treatment.

Taken together, these potential challenges not only highlight the limitations of current antiviral options but also reinforce the urgent need for continued innovation in antiviral drug development to ensure more effective and adaptable therapeutic approaches that can keep pace with viral evolution and better serve diverse patient populations.

Accurate monitoring of drug resistance mutations in the treatment of COVID-19 is crucial to alert individuals to potential reductions in drug efficacy due to mutations. Furthermore, identifying drug resistance mutations is essential for the development of newer, more potent drugs with reduced susceptibility to viral resistance.

## 6. Overview of Antiviral Candidates for COVID-19

Despite the great efficacy of approved clinical drugs against SARS-CoV-2, there are still limitations regarding their use, and with the emergence of variants that may be resistant to approved therapies, new studies are needed on the discovery of antiviral drugs that can improve the treatment of COVID-19. In this context, several antivirals have been developed in recent years, with the aim of improving treatments against COVID-19 and avoiding the mutations that arise in the new variants. Here, we have selected a range of antivirals that act at different stages of the SARS-CoV-2 replication cycle and are in different preclinical and clinical stages for the treatment of COVID-19 (Table 2).

### 6.1. Promising Inhibitors of Viral Entry

A significant portion of the current therapeutic research targeting SARS-CoV-2 has emphasized the S glycoprotein, which, once the RBD interacts directly with the human ACE2 receptor, constitutes a major target of the immune response during natural infection, which makes it an attractive component for therapeutic intervention [108]. Although several mAbs that were originally generated against SARS-CoV, such as CR3022 and S309, exhibited some level of cross-reactivity with SARS-CoV-2 RBD [109,110], most of the antibodies do not show cross-reactivity with this region [111]. The use of neutralizing mAbs, such as Bamlanivimab (LY-CoV555), as monotherapy or in combination with etesevimab, has produced positive clinical results by reducing the viral load of patients with early-stage, mild-to-moderate COVID-19 [84,85].

Other neutralizing mAbs, such as amubarvimab and romlusevimab (amubarvimab/romlusevimab), have been used since their approval at the beginning of the COVID-19 pandemic and are commonly recommended in China as antiviral treatment for adult patients infected with SARS-CoV-2 or those with a high risk factor for progression to severe COVID-19 [86,87]. These mAbs bind to distinct epitopes of the SARS-CoV-2 S protein, preventing internalization of the virus by its receptor without any interaction between them, which suggests good efficacy in the use of amubarvimab/romlusevimab [86]. More recently, in a study of post-COVID patients, it was demonstrated that treatment with a combination of neutralizing mAbs (amubarvimab/romlusevimab) was effective in preventing hospitalizations and deaths, but was not able to reduce the risk of long COVID [88]. Despite showing good effects during the initial years of the COVID-19 pandemic, the use of anti-SARS-CoV-2 mAbs for the treatment of COVID-19 is no longer recommended, since amubarvimab/romlusevimab is no longer in clinical development due to its lack of activity against the current circulating variants and the high mutation rate that these variants present in the S protein [88].

In addition to the RBD antibodies, the NTD of the SARS-CoV-2 S protein has also gained attention as a critical target for mAbs development, particularly due to its structural role and immunogenicity [112]. Notably, the 4A8 antibody demonstrates potent neutralizing activity by stabilizing NTD conformation and facilitating the proper arrangement of its loop structures, and, when used in conjunction with RBD-directed antibodies, it may contribute to a broader and more resilient therapeutic strategy by limiting viral escape [113]. Three other NTD-specific antibodies, CoVIC-247, CoVIC-245, and CoVIC-020, have also been identified and categorized as NTD-1 through NTD-3, respectively [89]. However, the continuous emergence of SARS-CoV-2 Omicron subvariants, which exhibit more than 30 mutations in the S protein compared to the original strain, has significantly impacted the efficacy of existing antibody-based therapies, leading to widespread immune evasion and reduced neutralization by previously effective antibodies [114,115,116]. Consequently, several mAbs once authorized by the U.S. FDA, such as bebtelovimab, sotrovimab, bamlanivimab (alone or with etesevimab), amubarvimab/romlusevimab, and the REGN-COV2 combination (casirivimab and imdevimab), are no longer recommended due to diminished activity against newer variants [88,117].

The current antiviral strategies against SARS-CoV-2 have largely relied on repurposing already approved drugs or screening extensive libraries of small molecules, including those at clinical stages or with FDA approval. Despite advances in mAb therapies, no small-molecule drugs that specifically target the S protein have been approved for COVID-19 treatment, which highlights a critical gap in antiviral development. To address this, various research groups have focused on identifying and optimizing S-specific small molecules. A high-throughput screen of over 15,000 compounds led to the identification of calpeptin, a potent inhibitor that interferes with SARS-CoV-2 entry and demonstrated activity against non-Omicron variants [90]. Similarly, sertraline, commonly known as an antidepressant, showed antiviral properties by binding to the RBD of the S1 subunit, thereby disrupting the S protein, its interaction with the receptor ACE2, and the necessary proteolytic processing of the S protein [91]. This molecule also blocks the viral entry of pseudotyped variants such as Omicron BA.1. Another molecule, arbidol, also known as umifenovir, that is used to treat influenza in China was demonstrated to inhibit SARS-CoV-2 by preventing S-protein trimerization, thus impairing viral attachment and fusion; recent clinical trials demonstrated its effectiveness in shortening the recovery time and enhancing the viral clearance in patients infected with Omicron [92,93].

### 6.2. Promising Inhibitors of Mpro

Although only a few cases of resistant SARS-CoV-2 following the use of nirmatrelvir have been reported, their potential emergence remains a major concern regarding this drug. For this reason, the development of Mpro inhibitors that target novel sites is needed, so that nirmatrelvir resistance can be overcome while reducing the risk of future evolutionary mutations that may emerge after continued treatment [94,95].

ISM3312 is an irreversible covalent Mpro inhibitor that has demonstrated a potent antiviral efficacy against multiple human coronaviruses, including SARS-CoV-2 and its VOCs. This antiviral drug forms a carbon-sulfur bond with Mpro, interacting with the active site of the enzyme and thereby blocking viral replication. Unlike nirmatrelvir, the binding site of ISM3312 may be less prone to developing resistance mutations since serial in vitro passage experiments did not reveal any mutations in the region in which ISM3312 binds with Mpro. Furthermore, ISM3312 inhibited viruses that were resistant to nirmatrelvir, and the sequential administration of ISM3312 with nirmatrelvir effectively mitigated the likelihood of viral resistance mutations of Mpro. ISM3312 exhibits low off-target risk because it does not require any pharmacokinetic boosters and can be orally administered. Therefore, ISM3312 is a promising broad-spectrum inhibitor against coronaviruses and a strategic backup candidate for potential future coronavirus infections [94].

AVI-4516 and AVI-4773 are uracil-based and nonpeptidic Mpro inhibitors that target additional Mpro subsites and use a latent electrophile to engage Cys145 irreversibly, both in vitro and in vivo. It was demonstrated that these compounds have pan-coronavirus antiviral activity, like that of ISM3312, and low clearance in mice. AVI-4773 has rapidly reduced viral titters after three doses and has been observed with 90% effectiveness after 8 h of oral administration. On the other hand, AVI-4516 has shown minimal inhibition of cytochrome P450 and has exhibited synergy with molnupiravir in cellular infection models. Both compounds have been able to inhibit the nirmatrelvir-resistant Mpro mutant virus and were well distributed into mouse tissues. These chemotypes are differentiated from existing clinical and preclinical Mpro inhibitors, and they can potentiate therapeutic development against emerging variants of SARS-CoV-2 [95].

An innovative Chinese oral drug was conditionally approved in 2023 for the treatment of mild and moderate COVID-19, becoming the first anti-SARS-CoV-2 drug with intellectual property rights in China. The drug was developed by the Shanghai Institute of Materia Medica, Chinese Academy of Sciences, Wuhan Institute of Virology, and Simcere Pharmaceutical Group Limited. This drug is called Xiannuoxin and features a combination of simnotrelvir and ritonavir [96]. Simnotrelvir is a small molecule that targets Mpro, an enzyme that is essential for SARS-CoV-2 replication, and ritonavir, which, at low doses, is used to slow the metabolism of simnotrelvir. Furthermore, it demonstrated safety and efficacy in phase II/III multicenter clinical trials that involved 1208 adult patients with mild-to-moderate COVID-19 infection [97]. Currently, Xiannuoxin is the most widely used antiviral for the treatment of COVID-19 in China, and its cost of treatment is around 25% of that of Paxlovid, which may be a factor for this product to find a market in other countries [96].

The oral antiviral drug RAY1216, also known as leritrelvir, was developed by Guangdong Zhongsheng Pharmaceutical and received conditional marketing approval from the National Medical Products Administration of China in March 2023 for the treatment of COVID-19 in adults with mild-to-moderate viral symptoms under medical supervision. The mechanism of action of leritrelvir specifically targets the main protease Mpro of SARS-CoV-2, preventing the cleavage of viral precursor proteins and consequently impeding viral replication. It has demonstrated antiviral effectiveness against the novel coronavirus, demonstrating strong efficacy in preclinical studies and exhibiting favorable safety profiles at various dosages in a phase 1 clinical trial [98]. In a randomized, double-blind clinical trial involving 60 adults with COVID-19 during the period of Omicron variant prevalence, both RAY1216 alone and in combination with ritonavir effectively reduced SARS-CoV-2 replication, significantly accelerating the viral clearance compared with placebo within 5 days after viral detection [99]. In a recent phase 3 clinical trial, leritrelvir without ritonavir boosting significantly reduced the time to recovery for mild-to-moderate symptoms of COVID-19 compared with placebo [100]. The favorable safety profile of this drug and its good efficacy in reducing the viral load of the Omicron variant suggest a promising future in the treatment of SARS-CoV-2 infections and their variants.

Apixaban, a drug with anticoagulant activity, was able to inhibit the replication of SARS-CoV-2 in vitro, acting directly on Mpro, an enzyme that is essential for the maturation of viral proteins. This effect occurred through a non-competitive mechanism, where the drug could not compete with the substrate for binding to the enzyme’s active site, which suggests an allosteric regulation mechanism. In vitro treatment of calu-3 cells with apixaban significantly reduced the viral load without inducing cytotoxicity, which indicates safety at clinically achievable concentrations. These findings suggest that the drug may play a dual therapeutic role in patients with COVID-19: in addition to preventing thromboembolic complications, common in severe cases of the disease, it may also act directly to reduce viral replication [101].

### 6.3. Promising Inhibitors of PLpro

The SARS-CoV-2 PLpro is a cysteine protease and a domain within NSP3. PLpro, along with Mpro, is responsible for the polyprotein process and is essential for viral replication. In addition, PLpro acts as a deubiquitinase and deISGylase, suppressing the host immune response [102]. In this context, PF-07957472 is an oral antiviral drug that binds irreversibly to the active site of the PLpro, forming a covalent bond with the cysteine residue (Cys111). It has shown potent cellular antiviral activity and efficacy in a mouse-adapted COVID-19 infection model [102]. Meanwhile, WEHI-P is a novel chemical scaffold that targets SARS-CoV-2 PLpro with low nanomolar activity and is efficient against other pathogenic coronaviruses. It has shown efficacy in a mouse model of severe acute COVID-19 and protection against a range of symptoms associated with the long-term sequelae of the disease, as well as preventing lung pathology and reducing brain dysfunction. Therefore, differently from other antiviral drugs, PLpro may have clinical relevance for preventing the long-term pathologies associated with COVID-19 [103].

### 6.4. Promising Inhibitors of RdRp

A new antiviral drug that has been developed in China, VV116 (Deuremidevir), a nucleoside analogue antiviral drug, has emerged as a promising oral treatment against SARS-CoV-2. It was jointly developed by the Wuhan Institute of Virology, the Shanghai Institute of Materia Medica, and other Chinese institutions [104]. It inhibits viral replication by non-covalently binding to the active site of the virus’s RdRp, a mechanism that was validated in preclinical models using both the original SARS-CoV-2 strain and key variants, including Omicron [105]. Robust clinical evidence has reinforced the potential of VV116. A phase III clinical trial demonstrated that the drug not only reduced the time to the sustained resolution of clinical symptoms but also demonstrated more favorable virological outcomes compared to placebo [106,107]. In recognition of these results, China’s National Medical Products Administration granted conditional approval to VV116, positioning it as a notable addition to the country’s antiviral arsenal against COVID-19 [104].

## 7. Conclusions and Future Perspectives

The development and implementation of antiviral therapies against SARS-CoV-2 have played a key role in reducing the global impact of COVID-19, contributing to reduced disease severity, hospitalization rates, and mortality. In this context, one of the most pressing challenges in the ongoing management of COVID-19 is the emergence of SARS-CoV-2 variants, which continue to impact the effectiveness of available therapeutic strategies. Viral evolution, particularly mutations in the S protein and other genomic regions, can alter the virus’s susceptibility to antiviral agents and compromise their clinical efficacy [63]. While direct-acting antivirals maintain activity across many variants due to their conserved targets in the viral replication machinery, the clinical effectiveness of some mAbs has been significantly reduced by S mutations [31].

Currently approved antiviral agents target distinct stages of the SARS-CoV-2 replication cycle and employ different mechanisms of action. Among them are RdRp (NSP12) inhibitors, such as remdesivir (Veklury), which is administered intravenously [5], and molnupiravir (Lagevrio), which is administered orally [55]. Another important treatment is nirmatrelvir/ritonavir (Paxlovid), a protease inhibitor combination that targets the Mpro and blocks the cleavage of the viral polyprotein precursors, and which is also administered orally [4,42]. These agents have demonstrated clinical efficacy in reducing viral load, preventing disease progression, and reducing hospitalization and mortality rates when used early in the course of infection.

Despite their benefits, each antiviral has specific limitations and potential adverse effects that must be carefully considered in clinical practice. Remdesivir has been associated with elevated liver enzymes (AST and ALT); therefore, its use is not recommended in individuals with severe hepatic impairment [53]. Molnupiravir can cause gastrointestinal symptoms such as diarrhea and nausea, and dizziness, and is contraindicated during pregnancy due to potential risks to the fetus [58]. Paxlovid^®^ has been associated with dysgeusia and diarrhea and has a higher risk of drug interactions compared to other antivirals, which limits its use in patients taking certain medications [41,45]. Furthermore, it is contraindicated in patients with severe renal or hepatic impairment.

One of the most pressing concerns in the therapeutic management of COVID-19 is the potential emergence of antiviral resistance. As the use of antiviral agents becomes more widespread, particularly in the form of monotherapy, there is an increasing risk that selective drug pressure may favor the emergence of resistant viral strains. Although resistance-associated mutations remain relatively uncommon in clinical settings, a growing body of in vitro evidence demonstrates that SARS-CoV-2 can acquire mutations that significantly reduce the virus’s susceptibility to key antiviral agents. For example, several mutations in the Mpro have been shown to confer resistance to nirmatrelvir, especially recurrent substitutions at E166, which are associated with markedly increased IC50 values. Other single-point mutations, including S144, M165, H172, and Q192, can also compromise nirmatrelvir’s inhibitory activity [64]. Moreover, combinatorial mutations, such as L50F/E166V, have demonstrated even higher levels of resistance than isolated substitutions [65,66,67]. Notably, mutations such as P132H and K90R, although located outside the active site, have been found to affect the conformational flexibility and structural dynamics of Mpro, and thereby indirectly reduce the efficacy of antivirals [67,70].

On the other hand, mutations in the RdRp (NSP12), the target of remdesivir, are less frequently observed but remain clinically relevant. Substitutions such as V166L, E802A, E802D, G671S, F480L, V570L, S759A, V792I, and C799F have been implicated in reduced sensitivity to remdesivir or its parent nucleoside GS-441524 [71,73]. Importantly, resistance to remdesivir tends to arise primarily after serial viral passaging under drug pressure, which suggests that many of these mutations could emerge from natural viral evolution rather than direct antiviral selection. In the case of molnupiravir, resistance-associated mutations have not yet been definitively identified. However, continued surveillance remains essential, particularly due to concerns that the drug’s mechanism of inducing mutagenesis could theoretically promote the emergence of transmissible variants, especially in immunocompromised individuals with prolonged infections. In this context, the A716V mutation in NSP12, found within the catalytic domain, has been detected in some treated patients, although its functional impact on drug resistance remains unclear [83].

Given these risks, it is increasingly clear that the combination of antiviral therapies that target multiple stages of the viral life cycle may offer a more effective strategy to suppress replication and reduce the probability of resistance development. While large-scale vaccination campaigns and improved clinical management have significantly reduced severe COVID-19 outcomes, SARS-CoV-2 continues to evolve, challenging the durability of current interventions. This reality highlights the ongoing need for novel antivirals, as well as the repositioning of existing drugs, to remain ahead of viral adaptation and immune escape. Ultimately, complementary antiviral strategies will continue to play a vital role in reducing the disease’s progression, controlling its transmission, and safeguarding the global health response against COVID-19 and future viral threats.

So far, resistance to molnupiravir has not been clearly defined, although ongoing studies are essential to monitor emerging variants under therapeutic pressure. However, some concerns have been raised about the potential for molnupiravir to promote the emergence of transmissible variants, particularly in immunocompromised hosts who may experience prolonged infection and incomplete viral clearance. In this context, the A716V mutation in RdRp has been detected in some patients receiving molnupiravir. Although this residue lies within the catalytic core of RdRp, no functional evidence has confirmed its role in drug resistance [83]. Overall, antiviral resistance represents a growing concern in the management of COVID-19, which emphasizes the importance of continued genomic surveillance and functional studies. These efforts are crucial to safeguard the efficacy of current treatments and guide the development of more robust future antiviral strategies.

**Figure 2:**
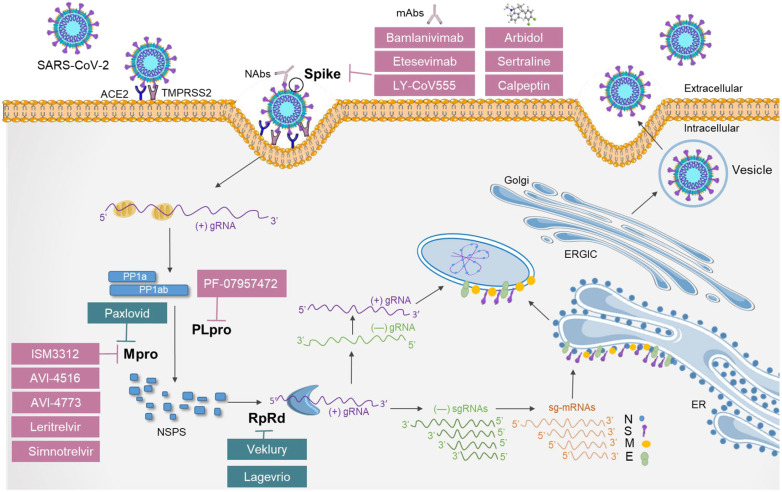
Targeting the SARS-CoV-2 life cycle: clinical and preclinical antiviral strategies. Schematic representation of the key antiviral drugs and their respective targets in the SARS-CoV-2 life cycle. Antiviral drugs highlighted in blue have completed clinical trials and are commercially approved for COVID-19 treatment. Antiviral drugs in purple are currently in phase 2/3 clinical trials or preclinical development, but the results show a promising potential for COVID-19 treatment.

Despite the demonstrated efficacy of currently approved antiviral therapies against SARS-CoV-2, important limitations remain, particularly in the context of emerging viral variants that may exhibit reduced susceptibility to these agents. These challenges underscore the urgent need for the continued development of novel antiviral compounds that can improve COVID-19 treatment and counter resistance associated with viral evolution. In recent years, considerable progress has been made in the discovery and preclinical and clinical evaluation of antivirals that target multiple stages of the SARS-CoV-2 life cycle, with the goal of reducing disease severity (Figure 2).

Much of the early therapeutic focus was directed toward the S glycoprotein, particularly its RBD, which directly engages the human ACE2 receptor and serves as a major immunological target during infection. This made the S protein a compelling candidate for antiviral interventions [108]. However, the high mutation rate within the S, especially in circulating variants, has led to a loss of the efficacy of mAbs, such as the amubarvimab/romlusevimab cocktail, which is no longer in clinical development [88]. Moreover, no small-molecule inhibitors that specifically target the S protein have been approved to date, which highlights a significant gap in therapeutic strategies. To address this, ongoing efforts are focused on the identification and optimization of S-specific small molecules that can maintain their efficacy across diverse viral lineages.

Although cases of resistance to nirmatrelvir, the main component of Paxlovid, remain rare, their potential emergence continues to raise concerns. Consequently, there is a growing interest in designing new Mpro inhibitors that target alternative binding sites to preserve therapeutic efficacy and minimize the likelihood of the development of future resistance [94,95]. In parallel, the PLpro, a domain within NSP3, has gained attention as a promising antiviral target. PLpro not only plays an essential critical role in polyprotein processing for viral replication but also antagonizes host immune responses through its deubiquitinase and deISGylase activities [103].

Furthermore, advances have been made in the development of RdRp inhibitors, which act by non-covalently binding to the RdRp active site to block viral RNA synthesis. This mechanism has been validated in preclinical studies that involved both the ancestral SARS-CoV-2 strain and major variants, including Omicron [105]. These emerging therapies, targeting conserved and functionally essential viral proteins, represent an important step toward broadening the antiviral arsenal and enhancing preparedness against future variant-driven outbreaks. Therefore, integrating optimized antiviral regimens with vaccination efforts will be necessary to achieving lasting control of the pandemic and anticipating future viral threats.

## Figures and Tables

**Table 1:** Antiviral therapies approved for COVID-19.

Antiviral Drug	Administration	Age Group	Therapeutic Target	Main Side Effects	Contraindications
Paxlovid (nirmatrelvir/ritonavir)	Oral	≥12 years who weigh at least 40 kg	Mpro	Dysgeusia, diarrhea, and drug-drug interactions	Severe hepatic and renal impairments
Veklury (remdesivir)	Intravenous	≥28 days who weigh at least 3 kg	RdRp	Elevation of liver enzymes AST and ALT	Severe hepatic impairments
Lagevrio (molnupiravir)	Oral	Adults with mild-to-moderate COVID-19 over 18 years old	RdRp	Diarrhea, nausea, and dizziness	Pregnancy

**Table 2:** Preclinical and clinical trials of antivirals for COVID-19.

Antiviral Drug	Viral Target	Main Results	Current Trial Phase	Experimental Models	Administration	References
Bamlanivimab (LY-CoV555) and etesevimab	Spike protein	Monotherapy or in combination with etesevimab reduced the viral load in patients with early, mild to moderate COVID-19	Phase II/III clinical trials	-	Intravenous	[84,85]
Amubarvimab/romlusevimab	Spike protein	Effective in preventing hospitalizations and deaths, but could not reduce the risk of long COVID	Phase III clinical trials	-	Intravenous	[86,87,88]
CoVIC-247; CoVIC-245 and CoVIC-020	Spike protein	Demonstrates potent neutralizing activity by stabilizing NTD conformation	In silico	-	-	[89]
Calpeptin	Spike protein	Demonstrated interference with SARS-CoV-2 entry and activity against non-Omicron variants	In vitro	Vero E6 and HEK293T-ACE2 cells	-	[90]
Sertraline	Spike protein	Inhibiting viral entry, and reducing the inflammatory response and pulmonary damage	In vitro and in vivo	Vero E6, Caco-2 and HEK293T-ACE2 cells; Mouse model	Oral	[91]
Arbidol (umifenovir)	Spike protein	Shortening recovery time and enhancing viral clearance in patients with Omicron	Phase II/III clinical trials	-	Oral	[92,93]
ISM3312	Mpro	Blocks viral replication through interaction with the enzyme’s active site, with any mutation in the ISM3312 binding region with Mpro	In vitro and in vivo	Vero E6 and Huh-7-hACE2; Mouse model	Intragastrically	[94]
AVI-4516 and AVI-4773	Mpro	AVI-4773 reduced viral titers, and AVI-4516 with minimal cytochrome P450 inhibition. Inhibit the nirmatrelvir-resistant Mpro mutant virus	In vivo	Mouse model	Oral	[95]
Simnotrelvir	Mpro	It demonstrated safety and efficacy in multicenter clinical trials involving 1208 adult patients with mild to moderate COVID-19 infection	Phase II/III clinical trials	-	Oral	[96,97]
Leritrelvir (RAY1216)	Mpro	Antiviral efficacy against SARS-CoV-2 and safety profiles at various dosages, significantly reduced time to recovery	Phase III clinical trials	-	Oral	[98,99,100]
Apixaban	Mpro	Antiviral with dual biological activity against COVID-19, reducing thromboembolism and inhibiting viral replication with low cytotoxicity.	In vitro	Calu-3 cells	-	[101]
PF-0797472	PLpro	Protection against long-term sequelae of the disease, with low doses, preventing lung damage and brain dysfunction	In vivo	Mouse-adapted COVID-19 infection model	Oral	[102,103]
Deuremidevir (VV116)	RpRd	Inhibit viral replication of the original SARS-CoV-2 strain and the Omicron variants. Reduced time to recovery and more favorable virological outcomes	Phase III clinical trials	-	Oral	[104,105,106,107]

## Abbreviations

The following abbreviations are used in this manuscript:

ACE2	Angiotensin-converting enzyme 2
ALT	Alanine aminotransferase
AST	Aspartate aminotransferase
CDC	Centers for Disease Control and Prevention
COVID-19	Coronavirus disease 2019
COVs	Coronaviruses
CYP450–3A4	Cytochrome P450 isoenzyme A
DDI	Drug–drug interactions
E	Envelope protein
EMA	European Medicines Agency
ER	Endoplasmic reticulum
ERGIC	ER–Golgi intermediate compartment
FDA	Food and Drug Administration
gRNA	Genomic RNA
KI	Kinase inhibitors
M	Membrane protein
mAbs	Neutralizing monoclonal antibodies
Mpro	Viral main protease
N	Nucleocapsid protein
NHC	β-d-N4-hydroxycytidine
NSP	Nonstructural proteins
NTD	N-terminal domain of RBD
NTP	Nucleoside triphosphate
ORF	Open reading frames
PLpro	Papain-like protease
RBD	Receptor-binding domain
RBM	Receptor-binding motif
RdRp	RNA-dependent RNA polymerase
RMP	Remdesivir monophosphate
RTC	Replication-transcription complex
RTP	Remdesivir triphosphate
S	Spike protein
SARS-CoV-2	Severe acute respiratory syndrome coronavirus 2
sgRNA	Subgenomic RNA
sg-mRNA	Subgenomic messenger RNAs
Tmax	Time of maximum plasma concentration of the drug
TMPRSS2	Transmembrane serine protease 2
VOC	Variant of concern
